# Supplementary material for: Inference via sparse coding in a hierarchical vision model
Source: J Vis. 2022 Feb 25;22(2):19. doi: 10.1167/jov.22.2.19 (PMC8883180; doi:10.1167/jov.22.2.19)
Supplement: Supplement 1 [file jovi-22-2-19_s001.pdf]

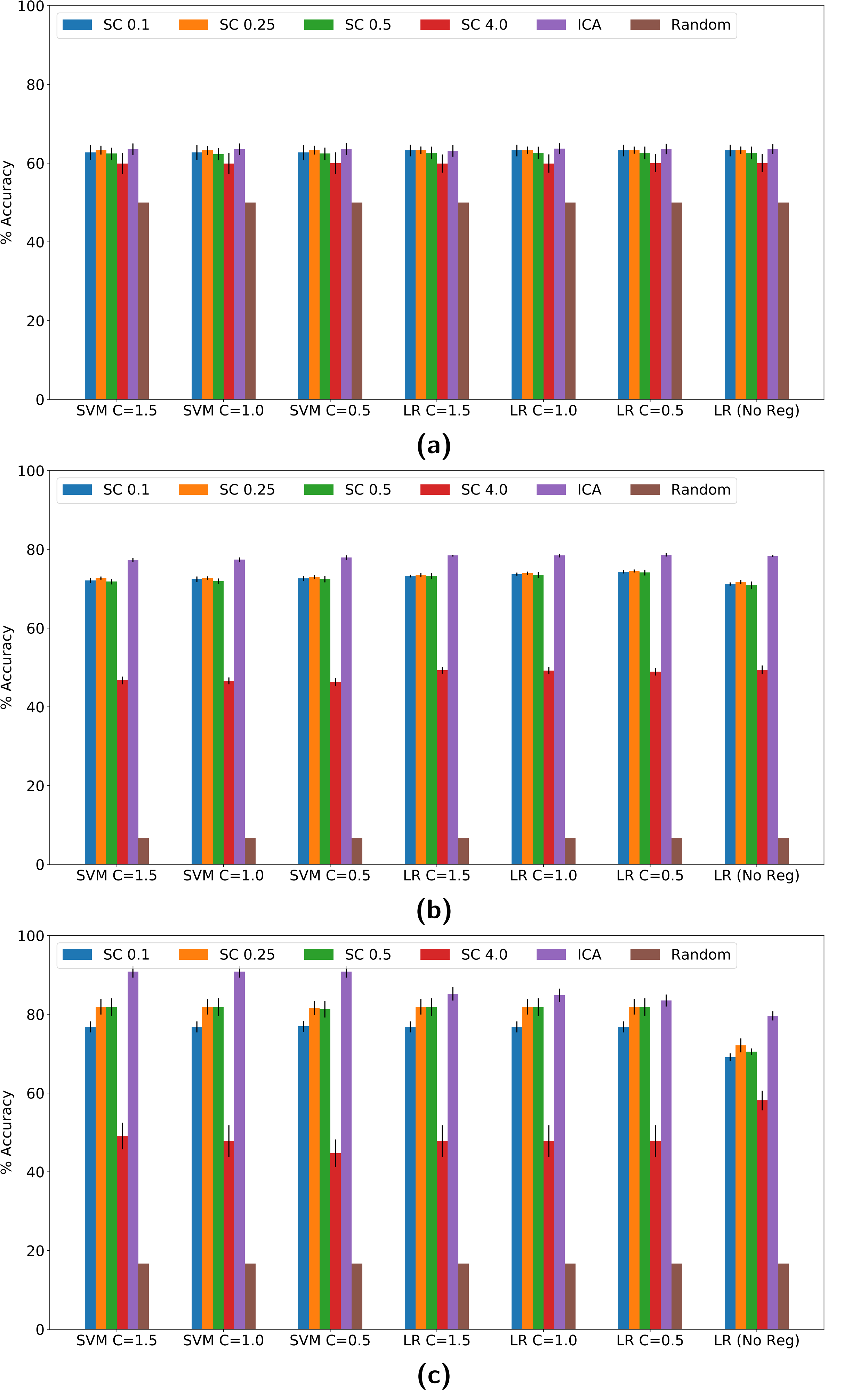

Figure 17: **Classification Results For All Models and Manipulations.** Average accuracy over 5-fold cross validation for non-negative sparse coding with regularization coefficients of 0.1, 0.25, 0.5 and 4.0 (listed as SC followed by the regularization coefficient) and overcomplete ICA (listed as ICA). Error bars reflect standard deviation over the 5 folds. Random denotes the result of guessing (expectation computed for the number of labels).
