# Supplementary material for: Inference via sparse coding in a hierarchical vision model
Source: J Vis. 2022 Feb 25;22(2):19. doi: 10.1167/jov.22.2.19 (PMC8883180; doi:10.1167/jov.22.2.19)
Supplement: Supplement 2 [file jovi-22-2-19_s002.pdf]

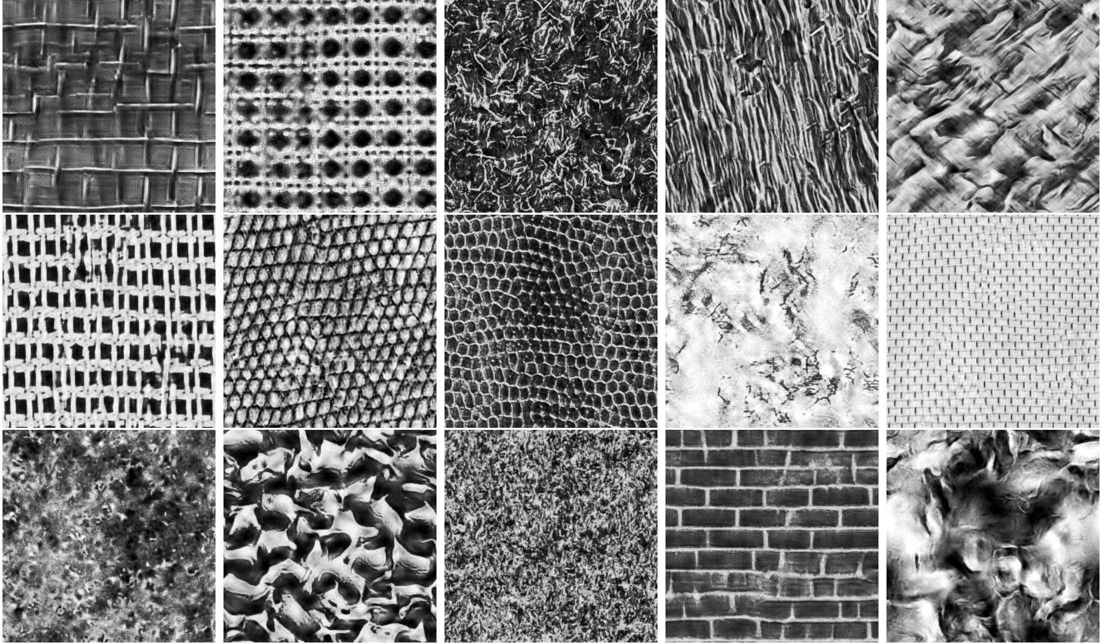

Figure 18: **Example 256x256 Texture Images Representing all 15 Classes.** These Brodatz texture images were generated with the code made available by Portilla and Simoncelli (2000). For each texture class 50 images were generated (making a total of 750 images). The 15 images shown here each represent one texture class. The 32x32 patches in figure 4 were randomly sampled from these images with an equal distribution across texture classes.

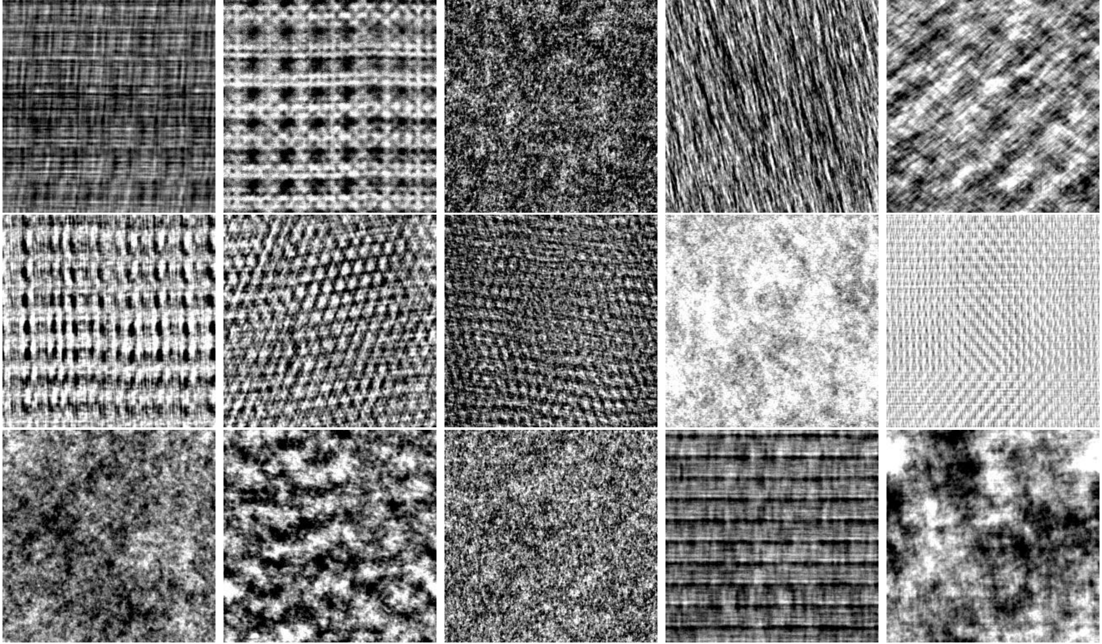

Figure 19: **Corresponding 256x256 Spectrally-Matched Noise Images.** Spectrally-matched noise versions of the textures (generated with the code of Portilla and Simoncelli (2000)) in figure 18. Each of the 750 full size texture images also has a spectrally-matched noise variant.
